# RECLAIM: Toward a New Era of Refurbishment and Remanufacturing of Industrial Equipment

**DOI:** 10.3389/frai.2020.570562

**Published:** 2021-02-15

**Authors:** Angeliki Zacharaki, Thanasis Vafeiadis, Nikolaos Kolokas, Aikaterini Vaxevani, Yuchun Xu, Michael Peschl, Dimosthenis Ioannidis, Dimitrios Tzovaras

**Affiliations:** ^1^ Information Technologies Institute, Center for Research and Technology Hellas, Thessaloniki, Greece; ^2^ College of Engineering and Physical Sciences, Aston University, Birmingham, United Kingdom; ^3^ Harms and Wende GmbH and Co., Hamburg, Germany

**Keywords:** refurbishment and remanufacturing, decision support framework, *in situ* repair, big data analytics, predictive analytics, industry, machine learning

## Abstract

Refurbishment and remanufacturing are the industrial processes whereby used products or parts that constitute the product are restored. Remanufacturing is the process of restoring the functionality of the product or a part of it to “as-new” quality, whereas refurbishment is the process of restoring the product itself or part of it to “like-new” quality, without being as thorough as remanufacturing. Within this context, the EU-funded project RECLAIM presents a new idea on refurbishment and remanufacturing based on big data analytics, machine learning, predictive analytics, and optimization models using deep learning techniques and digital twin models with the aim of enabling the stakeholders to make informed decisions about whether to remanufacture, upgrade, or repair heavy machinery that is toward its end-of-life. The RECLAIM project additionally provides novel strategies and technologies that enable the reuse of industrial equipment in old, renewed, and new factories, with the goal of saving valuable resources by recycling equipment and using them in a different application, instead of discarding them after use. For instance, RECLAIM provides a simulation engine using digital twin in order to predict maintenance needs and potential faults of large industrial equipment. This simulation engine keeps the virtual twins available to store all available information during the lifetime of a machine, such as maintenance operations, and this information can be used to perform an economic estimation of the machine's refurbishment costs. The RECLAIM project envisages developing new technologies and strategies aligned with the circular economy and in support of a new model for the management of large industrial equipment that approaches the end of its design life. This model aims to reduce substantially the opportunity cost of retaining strategies (both moneywise and resourcewise) by allowing relatively old equipment that faces the prospect of decommissioning to reclaim its functionalities and role in the overall production system.

## 1. Introduction

The industrial sector in Europe is very important as a “driver of sustainable growth and employment” (EP (2019)). High industrial productivity and efficiency are closely linked to well-functioning and well-maintained equipment, thus highlighting the critical role of machinery. However, not only in Europe is industry so vitally important for the economy. Currently, there is an estimated 40 billion dollars' worth of outdated machinery in use at US factories (M.NET (2016)), leading to an estimated loss of 50 billion dollars each year due to unplanned downtime resulting from machine failures (Studios (2018)). This is to some extent expected since many machines currently in use in production lines were installed well over 30 years ago and have exceeded their projected lifetime. In order to remain competitive, manufacturing companies should constantly improve the productivity and reliability of their production processes and equipment. In this perspective, maintenance activities have become even more crucial for business success. It is worth noticing that, nowadays, poor maintenance strategies reduce the industry's overall capacity between 5 and 20 percent (Wollenhaupt (2017)). This underlines the urgent need for improving the maintenance process, emphasizing the methods of refurbishment and remanufacturing. Refurbishment and remanufacturing are activities of the circular economy model, the purpose of which is to keep the high value of products and materials, as opposed to the currently employed economic model, thus targeting the extension of equipment and materials' life and reducing the unnecessary and wasteful use of resources. These two activities, along with health status monitoring, are the key elements for lifetime extension and reuse of large industrial equipment.

The EU Factory of the Future project, RECLAIM (*RE-manufaCturing and Refurbishment LArge Industrial equipment*), focuses on establishing and demonstrating technologies and techniques in order to support a new approach for refurbishment and remanufacturing of large industrial equipment in factories, setting forth the way to a circular economy. The project's main aim is to improve the maintenance process, emphasizing the methods of refurbishment and remanufacturing. Its ultimate objective is to preserve valuable resources by reusing equipment instead of discarding it. In this context, the project will develop new models and strategies for repairing and upgrading equipment and redesigning factory layouts to benefit the manufacturing sector from an economic perspective. These strategies are as follows: improving the machinery operation and avoiding unplanned downtime due to machine failure; estimating life cycle costs and contributing to the reuse of old machinery assets in renewed and new factories; providing maintenance able to identify equipment failures before they occur, in order to minimize the additional costs and downtime associated with the disassembly and transportation of the machinery and maximize the performance of the machinery during its lifetime.

The main scope of this work is to present the new paradigm for refurbishment and remanufacturing of large industrial equipment in factories, paving the way to a circular economy. In particular, firstly, we conducted a bibliographic review of the technologies, strategies, and tools that have been used to date to achieve the refurbishment and remanufacturing of the large industrial equipment in order to extend its lifetime. Then, the usefulness of the integrated technological solution RECLAIM was analyzed to prove that having RECLAIM technology available drastically increased efficiency, enhanced lifetime extension, and achieved high economic benefit and a significant step toward 100% reuse will be made. The added value of this article is to contribute to a better understanding of how the integration of RECLAIM technological solutions into industrial environments can lead to industries having an extra economic income from the extended lifetime of manufacturing systems and their components, which can be achieved by adopting refurbishment and remanufacturing solutions.

The rest of this article is organized as follows. In Section 2, the related work regarding key aspects that the RECLAIM project will face along with the proposed solutions dealing with these problems is analyzed, whereas the conceptual architecture is presented in Section 3. In Section 4, the main components of the architecture are described in full, and, finally, in Section 5, our conclusions are drawn.

## 2. Related Work and Proposed Solutions

In this section, in the beginning, the related works on key aspects of refurbishment and remanufacturing in the manufacturing environment are presented along with the ambition and solutions proposed by the project. The limitations of the RECLAIM platform are given at the end of the current section.

### 2.1. RECLAIM Solutions

The RECLAIM integrated architecture encompasses several modules and components, which are described in the following sections. These components are as follows: (a) decision support framework (DSF), (b) refurbishment and remanufacturing techniques for industrial equipment, (c) smart sensors’ network for industrial environments, (d) prognosis and health management, (e) cost analysis and cost modeling, (f) optimization planning on refurbishment and remanufacturing, (g) digital twin simulation engine, (h) cybersecurity for IoT devices, and (i) augmented reality (AR).

#### 2.1.1. Decision Support Framework of Used Industrial Equipment for Sustainable Manufacturing

Several methodologies and approaches for decision-making have been developed, addressing either single or multiple decisions, in order to enable the users and the process experts to assess the reusability or remanufacturability of production machines when they are facing the end of their production life. Ziout et al. (2014) provided a decision-making methodology for considering the end-of-life (EoL) product as a recovery option for all interested parties involved in the process. Thus, the decision taken is more accurate and more informative on the selection of appropriate recovery options. Remery et al. (2012) have provided an EoL scenario assessment methodology to evaluate the various solutions for the EoL scenario of a product during the early design phase, based on fuzzy techniques. Dhouib (2014) proposed a multicriteria decision analysis based on an extended (fuzzy) version of Measuring Attractiveness by a Categorical Based Evaluation Technique (MACBETH) methodology to take into consideration the imprecise and linguistic assessments provided by a decision-maker. Ovchinnikov et al. (2014) presented an analytical model and a behavioral study to demonstrate that remanufacturing frequently aligns the economic and environmental objectives of firms by increasing profits and reducing their overall impact on the environment. Ondemir and Gupta (2014) developed a multiple objective advanced order remanufacturing and disassembly (ARTODTO) system as an order-driven component and product recovery (ODCPR) system. The main objective of the proposed system was to evaluate whether a product needs remanufacturing, disassembly, repair, or recycling and to accomplish a variety of conflicting financial, environmental, and quality-based goals. Ng and Song (2015) introduced a method for determining the optimal for EoL product recovery options, using multiple factors of product condition and recovery values aiming to achieve better environmentally friendly decision-making regarding the maintenance, repair, or remanufacturability planning of the product. Dehghanbaghi et al. (2016) proposed an integrated approach, based on a fuzzy rule-based system and a fuzzy Analytic Hierarchy Process (AHP), to provide an accurate and valid decision-making mechanism to rank the recovery/disposal strategies. Kremer (2015) demonstrated a framework based on fuzzy logic and analysis that takes into account quantitative indicators (residual value and ecoindicators) and the social impact of each EoL option of the machinery.

Table 1 presents the key requirements for effective remanufacturing decision-making that are presented above compared to the aspects that RECLAIM DSF (*Decision Support Framework)* take into account.

**TABLE 1:** Key requirements for effective remanufacturing decision-making.

Author(s)	Key requirements for effective remanufacturing decision-making	Limitations
Ziout et al. (2014)	Recovery options for end-of-life products	Costs, environmental and health safety, durability, and viability of upgraded machine
Remery et al. (2012)	Fuzzy techniques during the early product design phase	Circular economy strategies
Dhouib (2014)	Extended (fuzzy) version of Measuring Attractiveness by a Categorical Based Evaluation Technique (MACBETH) methodology	Circular economy strategies, recovery options, durability, and viability of upgraded machine
Ovchinnikov et al. (2014)	Economic and environmental objectives	Circular economy strategies, recovery options, durability, and viability of upgraded machine
Ondemir and Gupta (2014)	Multiple objective advanced order remanufacturing and disassembly (ARTODTO) system as an order-driven component and product recovery (ODCPR) system	Durability
Ng and Song (2015)	Environmentally friendly decision-making	Circular economy strategies, recovery options, durability, and viability of upgraded machine
Dehghanbaghi et al. (2016)	Fuzzy rule-based system and a fuzzy AHP	Recovery options
Kremer (2015)	Quantitative indicators (residual value, ecoindicators, etc.) and the social impact	Circular economy strategies, recovery options, durability, and viability of upgraded machine

The aforementioned studies introduced several decision-making methodologies and frameworks that utilize fuzzy logic and include background of the product, environmental criteria, and stakeholders with the intention of providing a reasonable decision for the recovery or rejection of the product. Nevertheless, few of them could help to achieve both machine- and component-level health-based recovery planning while taking into account at the same time both environmental and economic reverberations. Thus, RECLAIM aims to develop a flexible recovery DSF to assist the machinery operators and machinery manufacturers in making efficient remanufacturing, repair, or rejection decisions at different service life periods on electromechanical machines and robotic systems. The proposed solution will combine inputs from optimization techniques, machine learning, digital twins, fault diagnosis, and predictive maintenance simulations.

#### 2.1.2. Uses of Refurbishment and Remanufacturing in Industrial Equipment

Refurbishment and remanufacturing can contribute significantly to well-being in Europe, as they are important lifetime extension strategies of resource-efficient manufacturing. In particular, the refurbishment process restores the system to meet its original specifications without replacing parts of the system (Varde et al. (2014)). Numerous methods have been used as refurbishment processes in a variety of industrial sectors (e.g., automotive, electrical, and electronics) in order to prolong the remaining useful life (RUL) of industrial infrastructure (Freiberger et al. (2011); Hatcher et al. (2013)). Atasu et al. (2008) have identified that the refurbishment process can be used as a productivity enhancement measure and as a marketing strategy. In this direction, Kerr and Ryan (2001) have reported that the refurbishment process reduces the total life cycle cost of the industrial infrastructure and is an ecofriendly method. However, many works have identified that the extension of the product life can also be achieved by using the remanufacturing process, which is also a value recovery option in order to extend industrial equipment's original lifespan (Chari et al. (2014)). In the work conducted by SteingrímssonPinar et al. (2011), the authors have introduced competitive business approaches regarding the remanufacturing market of production equipment. In a similar work, Cunha et al. (2011) have established a technology roadmapping methodology so as to portray the interconnections between market, equipment, and technology variables in the remanufacturing process. Moreover, a different make-to-order production strategy for automotive equipment was introduced by Schraven et al. (2012). In this work, the authors proposed a modular concept that enables consideration of recovered equipment components in engineering and design. Sharma et al. (2015) have explored the benefits of the remanufacturing process and showed that this process meets the requirements and needs of industries. In a more contemporary work, Darghouth et al. (2017) focused on the requirements of OEM perspectives for an effective remanufacturing process. Finally, the usefulness of the remanufacturing process was identified in many research works that were devoted either to machine tools (Ullah et al. (2016)) or to a special kind of production machines (Geng et al. (2016)).

With RECLAIM, a set of novel tools and methodologies will be developed for enhancing the refurbishment and remanufacturing processes for industrial electromechanical machines and robotic systems, differentiating the approach according to the level of action (whole machine, modules. and components). This approach is aiming not only to improve refurbishment and remanufacturing processes but also to participate in effective decision-making so as to achieve measurable performance improvements. The adaptive sensor network and digital retrofitting infrastructure will have the ability to be attached to machines, modules, and even components to be refurbished or remanufactured. This will allow legacy equipment to be a part of the IoT with advanced vertical and horizontal communication capabilities and also enable sophisticated data analysis, such as predictive maintenance. Those services will be supported by AR mechanisms (AR glasses) that will help technicians and manufacturers with a novel way to visualize and localize information on equipment refurbishment and remanufacturing operations directly situated on top of the physical equipment.

#### 2.1.3. Smart Sensor Network for Industrial Environment

Applications in the manufacturing domain, regarding real-time monitoring, maintenance planning, fault detection, etc., based on sensor network and data have yet to be widely adopted. Mourtzis et al. (2014b) focused on the integration of the customer into product personalization and aimed to support the design of manufacturing networks on the move through the development of apps for Android devices. Mori and Fujishima (2013) introduced a remote monitoring and maintenance system for machine tool manufacturers that uses a mobile phone. Tapoglou et al. (2015) adopted a cloud-based approach for monitoring the use of manufacturing equipment through a network of sensors dispatching assignments to designated computer numerical control machines and generating the optimum machining code. Teti et al. (2010) demonstrated that the primary requirements for sensorial monitoring systems in production involving sensors of any type (mostly vibration, acoustic, and temperature) are robustness, reconfiguration capability, intelligence, reliability, and cost-efficiency. Si et al. (2011) pointed out the increasing interest in the use of multiple sensors for condition monitoring and illustrated various multisensor data fusion methods and recent developments in diagnostics and prognostics of mechanical systems implementing condition-based maintenance. Rehorn et al. (2005) presented sensor and signal processing techniques used for tool condition monitoring systems in industrial machining applications. Moreover, D. Mourtzis et al. (2014) have proposed a framework of machine monitoring techniques for almost real-time machine status recognition that facilitates a predictive maintenance engine to minimize machine tool failures.

In order to provide automated knowledge extraction from big data gathered by the industrial partners, RECLAIM will use smart real-time control and data analytics, monitoring forecasted production lines and allowing prescriptive and preventative actions. RECLAIM will enable “just-in-time manufacturing” to continuously adjust to the business environment. The analytic tools will allow to easily adapt a) the quantity and variability of heterogeneous information sources across the factory life cycle, b) the different types of targeted manufacturing sectors and their structure, and c) the differences in the enterprise hierarchy level.

#### 2.1.4. Prognostics and Health Management Approach

To determine if a machine is worth refurbishing or remanufacturing, a Prognostics and Health Management (PHM) technique will be used for the estimation of the life cycle cost in relation to the maintenance activities of the machine. Many works have indicated that PHM techniques are used to reduce losses due to reliability issues (Pecht (2012)). Moreover, He et al. (2011) have successfully implemented the PHM techniques in the case of electromechanical migration on circuit boards, and more specifically, they proved that PHM techniques could be used in cases where the available physics-of-failure (PoF) models are unable to deliver satisfactory results, such as in Li-ion batteries and LEDs. Sun et al. (2012) have presented the challenges and benefits of the PHM techniques implementation and, more specifically, demonstrated that PHM techniques are based on the PoF approach, the data-driven approach, and the fusion approach. Toward this direction, Pecht and Jaai (2010) have reported that the physical understanding of the system failure mechanism, modeled mathematically, can determine the RUL. The main output of relevant research works has proved that machine learning techniques relying on the use of historical data can be classified as supervised and unsupervised learning techniques.

RECLAIM PHM will address the three main challenges concerning the refurbishment of a machine. First, is the machine worth refurbishing? Second, what is the best time to perform refurbishment at the least cost? Finally, how should the machine be refurbished? To determine if a machine is worth refurbishing, the RECLAIM implements well-established PHM techniques to estimate the life cycle cost associated with the refurbishment of the machine. If the cost of refurbishment is lower than that of a new machine, then it is a cost-effective option. In RECLAIM, the optimal time to perform refurbishment is determined by extracting a machine health indicator through PHM and estimating the RUL of the machine based on the indicator. Ideally, the machine should be refurbished close to the end of its life. To determine how the machine should be refurbished, *in situ* edge-monitoring of PHM provides diagnosis information so that the degradation levels of critical components are estimated in near real-time. The outcome of edge analysis is combined with machine specifications and historical trends to create a trustworthy RECLAIM DSF that can bring benefits in terms of reduced costs and the environmental footprint of manufacturing activities.

Therefore, detailed knowledge of the health status of the machinery and its components and the whole production line based on data of the RECLAIM PHM toolkit will offer peer-to-peer health evaluation of the machine and component predictive methods to increase asset uptime; RECLAIM will be able to identify the optimal time and the appropriate components for refurbishment or/and remanufacturing.

#### 2.1.5. Cost Analysis and Cost Modeling

Cost analysis and modeling cover the cost throughout the product life cycle, including design, manufacturing, service, and disposal. Manufacturing cost analysis and modeling have been well researched. Manufacturing costs normally include material costs, machining costs, and assembly costs, and the machining and assembly costs are normally calculated on the basis of the process design conducted by the production engineers (Xu et al. (2012)). The processing time can be determined based on the work rate of the resource used to perform each operation defined in the process. When the processing time is known, the machining or assembly costs can be estimated with the use of the cost rate of the resource utilization. For example, Xu et al. (2008a) adopted this approach to calculate the manufacturing costs in different applications, e.g., for aircraft life cycle cost modeling and automotive product manufacturing and remanufacturing cost modeling (Xu et al. (2014)). Maintenance costs are normally researched in the life cycle cost modeling covering different aspects. Xu et al. (2008b) developed aircraft life cycle cost modeling by using the Systems Engineering approach. In Xu and Feng (2014), a framework and cost model for the quantitative evaluation of the benefits of remanufacturing techniques to assist the decision-making on EoL strategies have been developed. Firstly, the additive manufacturing-based remanufacturing process has been modeled first; then, the cost breakdown structure for the process has been created; finally, the cost model has been developed.

Within RECLAIM, a real-time (or nearly real-time) accurate cost estimation model will be developed. This cost model will allow responsive and optimized decision-making regarding production and maintenance. This is achieved by conducting dynamic data collection based on the project's backbone big data infrastructure so that a real-time (or nearly real-time) accurate cost estimation can be carried out, which reflects the cost implications of real-time maintenance and projected disruptions to the production.

#### 2.1.6. Optimization Planning for Refurbishment and Remanufacturing

Refurbishment and remanufacturing activities in a finite planning horizon are discussed thoroughly in the scientific literature. In the work of Ferrer (1997), the complexity of personal computing remanufacturing and the difficulties in developing adequate recovery processes are addressed. Guide and Wassenhove (2001) have devised a framework for profitability estimation of reuse activities demonstrating the way the product returns management influences operational requirements and stating that the acquisition of used products may be used as a leverage mechanism for the management and productivity of reuse activities. An overview of quantitative models taking into account logistic environment issues arising in the context of reverse logistics was given in Fleischmann et al. (1997). In Fleischmann et al. (1997), the authors have proposed a heuristic solution methodology based on a mathematical programming model for the reverse distribution problem. This approach is attributed to the complexity of the proposed model. Pirkul and Jayaraman (1996) followed a similar approach, as they developed a mixed integer programming model to address the location issue with the objective to minimize the total transportation and distribution costs and the fixed costs for opening and operating plants and warehouses by employing Lagrangian relaxation to the model.

Although several researchers have suggested various remanufacturing models, fewer researchers have been able to address the problems in both business and strategic decision-making at the same time. In this project, some mathematical models for decision-making at both business and strategic levels in a remanufacturing environment are proposed. The functionality and the performance of the suggested models are thoroughly examined through computational experiments, simulation schemas of model parameters, and extensive data analysis from multimodal data sources.

#### 2.1.7. Digital Twin Simulation for Machinery Fault Diagnosis

The first attempt to analyze the concept of “twin” was made by NASA's Apollo Project in which researchers simulated the aircraft's twin body in a real physical system (Rosen et al. (2015)). In this way, astronauts could remotely observe the ship’s condition and make decisions for emergency situations (Wang et al. (2015); Negri et al. (2017)). Beyond this application of the solution, Tao et al. (2018) have demonstrated the usefulness of the digital twin model throughout a machine’s life cycle enabling the prediction of potential machine failures and closed-loop optimization of the machine design, production, operation, and maintenance. Moreover, in the same work, the authors have proved that the digital twin model allows technicians to verify the appropriate machine operation and monitor machine productivity. More specifically, they have demonstrated that the digital twin model can be used to control possible changes in the production phase before they are implemented since it could also be used to monitor the machine in all its operating phases and to reprogram it when necessary (e.g., from mass production to customized production). Finally, during the operation phase, the authors have affirmed that the digital twin model could be used as a verification tool for data collected by the factory on the performance of a product. Therefore, the digital twin model can offer value-added services with the support of physical simulation and data-driven intelligence. In more recent work, Magargle et al. (2017) have introduced a multiphysical twin model for monitoring the brake system status through multiple angles. To this end, the digital twin model can be implemented as a fault diagnostic service and as a RUL prediction and maintenance simulation tool, which can be utilized for prompt decisions and accident risk reduction.

In order to optimize maintenance activities (refurbishment, remanufacturing, etc.), RECLAIM will deploy a simulation engine that will create a virtual environment similar to the actual machine using the digital twin model. This model will analyze patterns in real-time and compare them with historical data about the machinery life cycle. The aim is to monitor and predict the performance and status of factory assets. In this manner, all the necessary information for the preparation and implementation of proper maintenance activities on the machines will be provided so that failures and production line stoppages are avoided. For this purpose, RECLAIM's digital twin model will be divided into three parts, i.e., the physical system (real industrial infrastructure), the digital system (simulated industrial infrastructure), and the data and information connection system that links the digital system with the physical system. RECLAIM's digital twin model saves time and money and helps reduce costly production downtimes as it predicts promptly possible failures in infrastructure.

**FIGURE 1:**
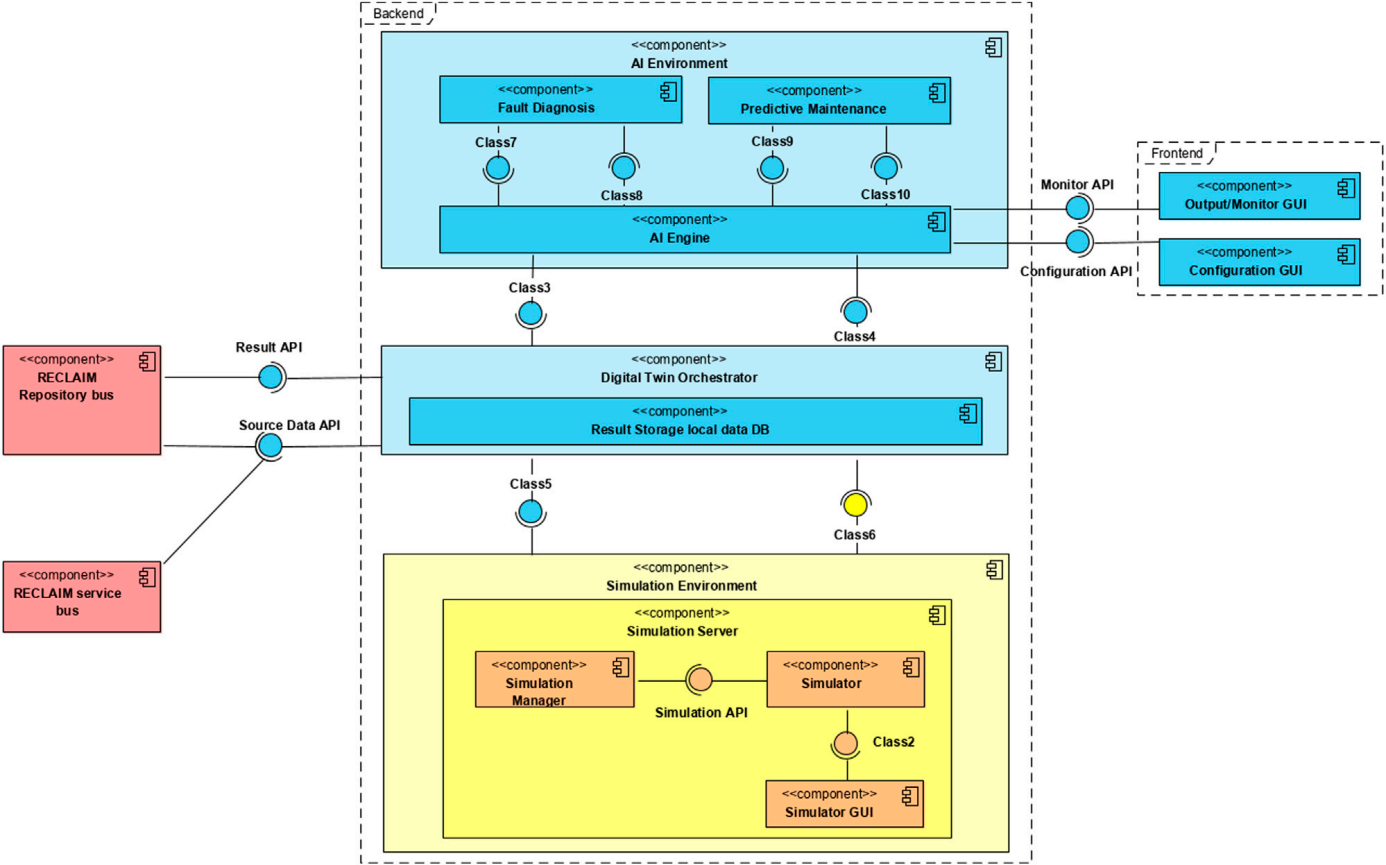
Fault diagnosis and predictive maintenance simulation engine using digital twin.

Digital twin component, given in Figure 1 includes the following subcomponents: a) the artificial intelligence (AI) environment, an engine leveraged to host and run the Fault Diagnosis and Predictive Maintenance algorithms; b) the AI engine, that is hosted in the AI environment and used to abstract the heterogeneous algorithms of Fault Diagnosis and Predictive Maintenance and to control their interactions; c) an orchestrator, that is used to orchestrate all tasks of the component, coordinating the interactions among AI engine and the distributed simulation environment, and receives the historical and real-time data, stores them, and processes them with data quality mechanisms; d) the simulation environment that is capable of running on different machines, each one wrapped by a simulation manager.

#### 2.1.8. Cybersecurity for IoT Devices for Connected Smart Environments

Poor usability of cybersecurity solutions tends to be the effect of security constraints. Finding the right trade-off between usability and security or the preferable integration of usability and security requirements is part of a major research challenge, which recently has been addressed by scholars (Realpe et al. (2015)). For instance, user-centered approaches are recommended as means to accomplish usable security, while the definition of objectives for both security and usability is suggested as a way to decide on the right balance between the two (Dhillon et al. (2016)). Understanding the security and usability collectively is recognized as a critical factor for the successful development, implementation, and usage of information systems (Andriotis et al. (2016)). As far as the IoT is concerned, usability and security are identified as two of the four major research challenges (the other two being performance and reliability); privacy concerns are growing, as IoT device manufacturers for smart homes are acquired by large corporations, such as Google (Alur et al. (2016)). The most recent research suggests new usable security frameworks, particularly for modeling security and privacy risks in smart homes at the consumer level. For example, the framework presented in Nurse et al. (2016) aims to support home users with a highly usable security decision support tool. However, it still needs to address improvements on usability and scalability and validate the real utility offered to the user.

Within this project, the edge-computing capabilities of the proposed RECLAIM Solution will be enhanced with lightweight security methods in order to empower resilience to cyberattacks and intrusion detection and prevention. This will allow shared access and flexibility in data governance for edge-based applications and reconfiguration and actuation. Moreover, in order to ensure seamless and trusted service provision over different data, the RECLAIM Solution will be enhanced with capabilities related to the dynamic coupling of microservices offered and embedded devices involved.

#### 2.1.9. Augmented Reality on the Plant Floor

AR is an emerging technology that can help manufacturers and maintainers, providing the necessary information which are needed regarding the maintenance/refurbishment/remanufacturing procedure by displaying virtual information on top of it. The main challenges faced by manufacturers or maintainers are as follows: (a) a large variety of tasks from diagnosis to repair; (b) varying complexity of maintenance requirements; (c) long life of equipment causing varying levels of quality, standards, and depth in documentation; (d) a large number of equipment types to maintain. It is noteworthy that AR offers opportunities for industrial maintenance applications by displaying contextualized information and accessing end-user data.

AR has received increasing attention from researchers in the manufacturing technology community as it is an interactive experience of a real-world environment and a technology that expands the physical world, adding on top of it layers of digital (virtual) information (Ong and Zhu (2013)). AR makes it possible for the user to gain information about a real-life process or procedure directly related to the work environment. This is the main coefficient factor for considering AR as an effective tool to be also used in through-life engineering services (Dini and Dalle (2015)). Several applications of AR in the industrial domain (maintenance, fault diagnosis, etc.) have been considered, but their research still remains at an exploratory level (Wang et al. (2016)). In order to study the effectiveness and the usability of AR integration in industrial fields (Oliveira et al. (2013)), new topics, such as authoring and context awareness, have arisen in this area (Erkoyuncu et al. (2017)). Authoring is a system component enabling the maintenance experts to develop, modify, and update applications’ AR contents (Gimeno et al. (2013); Roy et al. (2016)), whereas context awareness is a system using context to provide to the user task-oriented information and/or services (Manuri (2016)). These main properties of AR focus on how information regarding maintenance is acquired, transformed, and presented to the process experts and maintainers so that they decide instantly about further maintenance steps and assure that the process will not be fatally disturbed in any way.

The RECLAIM project aims at using AR techniques to support maintenance operations in the industrial domain by creating augmented features in real-time. More specifically, the RECLAIM AR-enabled multimodal interaction system will address the above challenges as it will provide a novel way to visualize and localize information on equipment refurbishment and remanufacturing operations directly situated on top of the physical equipment. During the refurbishment and remanufacturing operations, the system will also provide animated 3D stepwise instructions on disassembly and reassembly required, as well as support in the form of on-the-job remote assistance with real-time audio-visual communication and 3D annotation to technicians during the procedure. In this way, the variety of tasks from diagnosis to repair is addressed by showing the steps that the technician must follow to repair or maintain the machine. Also, information about the useful lifetime of the machine will be displayed as pop-up messages. At the end of the repair, a function registration message will appear that will take into account the different quality levels, standards maintained, etc. One more functionality of this solution is that the system itself performs the authoring steps that require AR expertise and maintainers have to indicate the key information for display, defining its format and sequence. The Authoring Platform is a human-machine interaction interface that allows maintainers to interact with the “Information Frameworks” used for creating AR features. The Application Platform Modules automatically generate AR features according to maintainers' feedback. Apart from contextualizing and rendering maintenance information, they ensure that the information is displayed in the right sequence. The developed system is versatile and effective regarding the support of the maintenance work of both novice and more experienced technicians.

### 2.2. RECLAIM Platform Limitations

Despite the numerous advances of the RECLAIM Solution, some possible limitations have been considered. First of all, some pilots had not stored any related data before the beginning of the project, so training machine learning models or statistical analyses cannot be done based on many data from the past. This will raise the need for estimations based on information found in the bibliography about similar equipment. Also, probably some data (e.g., end-user actions) cannot be provided automatically by some hardware, so the end-users will have to interact with the RECLAIM platform to insert them manually. Furthermore, due to the complexity of interdependencies among software components and the algorithm underlying each of these components, it is possible for computational delays to occur due to the communication latency and/or time complexity of algorithms. In addition, the memory aspect should be taken into account. Given the plenty of pilot raw data, corresponding to several machines and their physical components, and output data from numerous software components, it may be challenging for a single database to store all of them.

### 2.3. RECLAIM Framework Validation

Several pilot sites across Europe will be used for demonstration, evaluation, and assessment activities of the RECLAIM project. Due to confidentiality issues, the pilot sites will not be named here. The RECLAIM framework will be tested on a white goods company for (a) refurbishment and renovation of robot cells in B-Cell for making tubs (they reach the end of their life and have a large number of failures in their operation, unexpected stoppages, and delays in the production line); (b) modernization and refurbishment of an automatic spraying cabin, using enamel powder applied with help of spraying guns, in a shoe-making factory for maintenance and upgrade of cutting machines (prognostics and health assessment and predictive maintenance or refurbishment or remanufacturing methodologies in terms of production system efficiency, production cost, and product quality), in a wood manufacturing plant for predictive maintenance and refurbishment of the woodworking large production line (refurbish the system with additional sensor capable of monitoring product quality and identify the cause of deviations and predict failures and breakdown(s)), in friction welding machinery for lifetime extension by remanufacturing and predictive maintenance (lifetime prediction, remanufacturing of the machine in order to meet the today's requirements, continuous online monitoring of the machine's state, and predictive maintenance features), and finally in a bleaching machine for maintenance, refurbishment, and upgrading (holistic core process parameters optimization, monitoring and process control tool, a safe and stable operation, and improving the resource efficiency).

## 3. Conceptual Architecture

**FIGURE 2:**
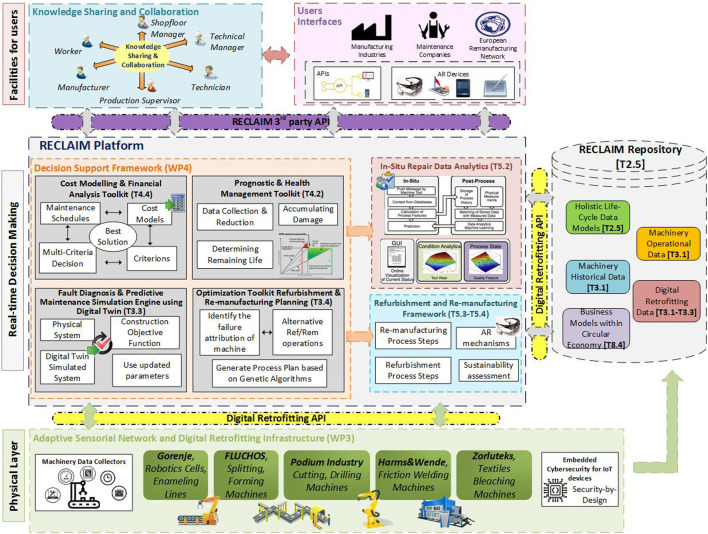
RECLAIM conceptual architecture.

Figure 2 presents the envisioned architecture of RECLAIM. The integrated architecture encompasses several modules and components, which are described in the following sections. The physical layer of the conceptual architecture will be supported by several manufacturing pilots providing the necessary and on-point business scenarios on industrial machine remanufacturing, refurbishment, and maintenance. The IoT infrastructure will be based on a three-stage design, where stage 1 comprises the existing wireless sensors (monitoring, smart object, environmental, and legacy), stage 2 includes sensor data aggregation systems enabling analog-to-digital data conversion, and in stage 3, edge IT functions and modules perform preprocessing on multimodal data before moving on to the data center for the main processing. Furthermore, the machinery operational and historical data, along with life cycle and business models and digital retrofitting data, will be collected and stored at the data repository for use by the data analytics algorithms. The core component of the proposed architecture is the RECLAIM platform, a dedicated environment that contains three key components: the *DSF,* the *in situ repair data analytics*, and the *refurbishment and remanufacturing framework*. Finally, the topmost part of the proposed conceptual architecture is the *User(s) Interfaces* (UI), along with the *manufacturing industries*, the *maintenance supportive companies,* and the *European Remanufacturing Network* (ERN).

### 3.1. Architecture's Main Components Description

As mentioned above, the key components of the RECLAIM platform are the *DSF*, the *in situ repair data analytics*, and the *refurbishment and remanufacturing framework*, which are described in detail as follows.

#### 3.1.1. Decision Support Framework

The *DSF* component is designed to support and improve the effectiveness of decisions concerning the refurbishment and remanufacturing of production infrastructure. The DSF will identify the most suitable actions/strategies based on different criteria such as the impact and the value of refurbishment or enhanced maintenance to extend asset life, the optimal time for replacing an asset, the machines' condition and possible upcoming failures, production planning, and resources allocation. The DSF will include novel tools as follows: the *Cost Modeling and Financial Analysis toolkit* (providing an effective cost estimation and financial impact analysis by using a combination of parametric and activity-based costing methods, while having the ability to take into account considerations of generality and reusability for the adaptation and uptake in wider industrial environments); the *Adaptive Sensorial Network and Fog Computing Framework* (providing information on the state of the machinery, such as temperature, operating speed, rotating speed, power consumption, torque, and vibration so as to minimize human interaction, increase mechanical automation, and identify pain points of machinery); the *Prognostic and Health Management toolkit* (providing a peer-to-peer health evaluation and component prediction methods to increase machine lifetime, productivity, and service quality); the *Fault Diagnosis and Predictive Maintenance Simulation Engine using Digital Twin* (creating a digital twin of the factory environment so as to use it to monitor and predict the performance and status of factory assets, while providing the user with all the features needed to schedule the maintenance works on the machines in order to avoid failures and to perform proper maintenance planning); the *Optimization Toolkit for Refurbishment and Remanufacturing Planning* (supporting the planning optimization through multivariable monitoring of the machine's operational parameters where the effects of variable changes will be possible to determine and combine best-known practices methodologies for model-based plant-site/shop-floor control). The proposed DSF will have attributes from both knowledge-driven and model-driven types of DSF based on the implementation of nondeterministic finite-state automata (finite number of states for the specified machine), simple scoring mechanisms, rule-based decision-making, and AI algorithms. Moreover, the implementation of data mining algorithms such as decision trees, genetic algorithms, and support vector machines will ensure the extraction of valuable information from IoT data. A *Visual Analytics Suite* will be built on the top of DSF in order to provide users with the most effective presentation of DSF output in the form of strategies, alternatives process models, KPIs visualization, and real-time health assessment of different production aspects.

#### 3.1.2. *In Situ* Repair Data Analytics

Industrial analytics are used to identify and recognize machine operational and behavioral patterns, make fast and accurate predictions, and act with confidence at the points of decision. Situational awareness as a mental state can be considered as a state of knowledge, which can be achieved using various techniques. In particular, in order to raise awareness of the health status of the machine and the situation of the shop floor during maintenance activities, data analytics techniques can be deployed. So, the knowledge about the situation of machines or/and shop floor can be gained from descriptive analytics (gain insight from historical or current data streams), predictive analytics (creation of predictive models utilizing statistical and machine learning techniques for the identification of machine and processes behaviors), and prescriptive analytics (finding optimal solutions based on descriptive and predictive analytics aspects). In addition, big data methodologies (e.g., visual analytics and visualization techniques) for the analytics of the big and diverse volume of available data gathered by the industrial partners also can give some valuable information about situational awareness. Visual analytics and advanced information visualization technologies can be exploited to present relevant information to different users (shop-floor manager(s), technician(s), manufacturer(s), etc.) in a user-friendly and effective manner. In particular, visual analytics has historically played a key role in business processes optimization. Existing tools can be of great assistance for the visualization of spatiotemporal data in the plant-site/shop-floor, providing temporal plots and heat maps indicating specific types of activities and representation of process time series data along with statistical analysis suitable to assist in discovering variable correlation. The proposed visual analytics suite will be developed based on two major stages so as to support situational awareness: perception (monitoring), which refers to the knowledge of the elements in the environment of plant-site/shop-floor, and comprehension (inspection and exploring), which refers to the combination and the integration of elements received by the sensors network. In terms of on-site repair analytics, both streaming analytics from the field of the repair (e.g., work process of repair) and batch analytics results based on on-demand queries will be applied in a planning time horizon so as to ensure enhancing human decisions and understanding and generating significant confidence in the final decision.

#### 3.1.3. Refurbishment and Remanufacturing Framework

Production planning, scheduling, and control of the fleet of an industrial ecosystem are major managerial challenges in the field of management operations in a manufacturing environment. A complete system approach is important to address all aspects of the production planning optimization, taking into account refurbishment and remanufacturing activities. This component aims to support the planning optimization through multivariable monitoring of the machine's operational parameters where the effects of variable changes will be possible to determine and combine best-known practices methodologies for model-based plant-site/shop-floor control. Based on the multimodal data provided by the IoT infrastructure, new approaches of real-time production planning optimization algorithms, from the perspective of machine learning techniques, will be researched and developed to apply proven optimization methodologies, provide the answers an end-user needs for effective decision-making, and consequently delivers measurable performance improvements. The data and information requirements are integral parts of the optimization phase. To create clear value from this information, production monitoring and surveillance is the first step in the measurement phase and is a prerequisite to analysis, improvement, and control. This monitoring might take into consideration the data collected from the *Adaptive Sensorial Network* together with recognition of any system constraints and behaviors.

### 3.2. Architecture's Core Innovations

RECLAIM is an ambitious project that will create and deploy an integrated DSF for machinery lifetime extension. The DSF for the optimization of refurbishment and remanufacturing process in itself is a significant step beyond the state of the art in the provisioning of infrastructure, tools, and services for experimentation in the digital manufacturing domain. The RECLAIM project will offer the following core innovations:

- *DSF* for refurbishment and remanufacturing optimization goes beyond the state of the art by allowing automatic and concurrent multiobjective (particularly for three or more objectives) decision-making and assessing multiobjectives in the same turn, so it provides efficient and optimized decision support.
- *In Situ Repair Data Analytics* for situational awareness will be a flexible tool, allowing the end-users to connect and integrate with any data source in real-time so as to have the ability for online visualization of significant KPIs for the current status of the machine (inspection) and the repair process. Condition analytics will be a key component that will combine state-of-the-art condition monitoring in order to go beyond machine health management.
- *Refurbishment and Remanufacturing Framework* will be enhanced with RECLAIM Solution having a sole objective; that is, the quality of remanufacturing/refurbishment process will have to follow strict reconditioning operations (steps). This effort will be supported by AR tools that utilize the sense of the worker, the ambient environment, and the context of work in the plant-site/shop floor.

## 4. Conclusion

The RECLAIM framework ensures that the remanufacturing and refurbishing interventions make a positive contribution not only toward business (i.e., increased return on investment, lifetime extension of the machinery, and alignment of its capabilities with the actual and future needs of the industry) but also toward the environment (i.e., improved material and resource efficiency and lower environmental impact). In particular, the proposed framework answers the following questions: *when* is the right time to refurbish or remanufacture industrial machinery, *what* is the appropriate strategy to follow, *which* benefits should the manufacturing company expect, and *how* this strategy will be implemented to deliver those benefits while providing enhanced reliability and safety of the refurbished or remanufactured equipment. The advanced RECLAIM framework aims to assist the machinery operators and machinery manufacturers in making efficient EoL decisions at different service life periods. This framework will consist of the following: a) the core RECLAIM toolkit (e.g., a Cost Modeling and Financial Analysis toolkit, a Prognostic and Health Management toolkit, a Fault Diagnosis and Predictive Maintenance Simulation Engine using Digital Twin, an Optimization Toolkit for Refurbishment and Remanufacturing Planning, and an *In Situ* Repair Data Analytics for Situational Awareness); b) a reference architecture along with a set of circular economy strategies and methodologies for manufacturing companies and OEMs.

RECLAIM is an ambitious project that will create and deploy an integrated DSF for machinery lifetime extension. The DSF for optimization refurbishment and remanufacturing process in itself is a significant step beyond the state of the art in the provisioning of infrastructure, tools, and services for experimentation in the digital manufacturing domain. The RECLAIM project will offer the following key novelty aspects: (a) a DSF for refurbishment and remanufacturing optimization by allowing automatic and concurrent multiobjective (particularly for three or more objectives) decision-making and assessing multiobjectives in the same turn, so it provides efficient and optimized decision support; (b) a Fault Diagnosis and Predictive Maintenance Simulation Engine using Digital Twin to keep the virtual twins that could store all available information during the lifetime of a machine, such as maintenance operations, and this information can be used to perform an economic estimation of the machine's refurbishment costs; (c) *In Situ Repair Data Analytics for Situational Awareness* that allows the end-users to connect and integrate with any data source in real-time so as to have the ability for online visualization of significant KPIs for the current status of the machine (inspection) and the repair process. Therefore, RECLAIM could answer *when* is the right time to refurbish/remanufacture industrial machinery, *what* is the appropriate strategy to follow, *which* benefits should the manufacturing company expect, and *how* this strategy will be implemented to deliver those benefits while providing for enhanced reliability and safety of the refurbished/remanufactured equipment. Through the proposed framework (DSF and associated tools, methodologies, and services), RECLAIM ensures that the remanufacturing and refurbishing interventions make a positive contribution not only businesswise (i.e., increased return on investment, lifetime extension of the machinery, and alignment of its capabilities with the actual and future needs of the industry) but also toward the environment (i.e., improved material and resource efficiency and lower environmental impact).

The next step after the implementation of RECLAIM is the validation of the proposed technology and the DSF through demonstrations. Those demonstrations will focus on large industrial equipment (e.g., industrial robotic systems, machines, AND production lines) from distinct industrial sectors: footwear manufacturers, white goods (cookers, dishwashers, etc.) manufacturers, wood manufacturing, friction welding machines, and textile manufacturers. Pilots will be supported by existing circular economy methods, which will be classified according to the preferable operation mode.

## Data Availability

The original contributions presented in the study are included in the article/Supplementary Material, further inquiries can be directed to the corresponding author.
